# Mitochondrial Quality Control in Inherited Mitochondrial Cardiomyopathy: Convergent Pathobiology and a Testable Therapeutic Framework

**DOI:** 10.3390/ijms27188270

**Published:** 2026-09-17

**Authors:** Chung-Lin Lee, Chih-Kuang Chuang, Ya-Hui Chang, Huei-Ching Chiu, Yuan-Rong Tu, Yun-Ting Lo, Jun-Yi Wu, Huang-Ying Huang, Hsiang-Yu Lin, Shuan-Pei Lin

**Affiliations:** 1 Department of Pediatrics, MacKay Memorial Hospital, Taipei 104217, Taiwan; clampcage@gmail.com (C.-L.L.); wish1001026@gmail.com (Y.-H.C.); g880a01@mmh.org.tw (H.-C.C.); 2 Institute of Clinical Medicine, National Yang-Ming Chiao-Tung University, Taipei 112304, Taiwan; 3 International Rare Disease Centre, MacKay Memorial Hospital, Taipei 104217, Taiwan; andy11tw.e347@mmh.org.tw (Y.-T.L.); wl01723138@gmail.com (J.-Y.W.); 4 Department of Medicine, MacKay Medical University, New Taipei City 252005, Taiwan; 5 Department of Nursing, MacKay Junior College of Medicine, Nursing and Management, New Taipei City 252005, Taiwan; 6 Division of Genetics and Metabolism, Department of Medical Research, MacKay Memorial Hospital, Taipei 104217, Taiwan; mmhcck@gmail.com (C.-K.C.); likemaruko@hotmail.com (Y.-R.T.); 7 College of Medicine, Fu-Jen Catholic University, New Taipei City 242062, Taiwan; 8 Department of Nursing, MacKay Memorial Hospital, Taipei 104217, Taiwan; hhy6543@mmh.org.tw; 9 Department of Medical Research, China Medical University Hospital, Taichung 404327, Taiwan; 10 Department of Infant and Child Care, National Taipei University of Nursing and Health Sciences, Taipei 112303, Taiwan

**Keywords:** mitochondrial cardiomyopathy, oxidative phosphorylation, cardiolipin, mitochondrial quality control, mitophagy, Barth syndrome, Friedreich ataxia, elamipretide, gene therapy

## Abstract

Inherited mitochondrial cardiomyopathies arise from pathogenic variants affecting oxidative phosphorylation, mitochondrial DNA maintenance, cardiolipin remodeling, protein import, cofactor metabolism, and mitochondrial dynamics or proteostasis. These disorders may be cardiac-predominant or part of multisystem disease. Their overlapping cardiac phenotypes suggest convergence on interacting pathways of energetic stress, cristae disruption, calcium imbalance, and redox injury, but do not establish a universal requirement for defective mitophagy. Mitochondrial quality control encompasses protein surveillance, membrane remodeling, dynamics, biogenesis, and organelle disposal; mitophagy is one component. We critically examine the hypothesis that inadequate clearance of damaged mitochondria contributes to progression in a subset of genotypes and disease stages. Disease-specific studies provide support in selected Barth syndrome models, whereas findings in frataxin deficiency vary with model and assay. We distinguish mitochondrial delivery to lysosomes, dynamic turnover measurements, and changes in pathway markers, and identify indirect evidence from acquired heart disease and fatty acid oxidation deficiency. Therapeutic evidence is separated into cellular, animal, and human studies and approved indications. Elamipretide has accelerated approval for muscle-strength improvement in patients with Barth syndrome weighing at least 30 kg; cardiac disease modification remains unconfirmed. Gene replacement has reached early clinical testing, including adeno-associated virus-mediated frataxin gene delivery (AAV-FXN), whereas mitochondrial genome editing and selective mitophagy modulation remain investigational. We propose testable predictions addressing progression, selective rescue, and treatment timing, together with outcomes that would challenge the hypothesis. This framework supports genotype- and stage-specific investigation without assuming that enhanced mitophagy will benefit every mitochondrial cardiomyopathy.

## 1. Introduction

Inherited mitochondrial cardiomyopathy presents a clinical paradox: diverse genetic defects produce overlapping myocardial phenotypes, yet the same pathogenic variant can be associated with very different timing and severity of cardiac disease. Here, the term denotes structural or functional myocardial disease caused by a primary inherited mitochondrial defect, either as a cardiac-predominant presentation or, more commonly, within a multisystem disorder. The initiating lesion may involve oxidative phosphorylation (OXPHOS), mitochondrial DNA (mtDNA) maintenance, protein import, cardiolipin remodeling, dynamics, or proteostasis; persistent adenosine triphosphate (ATP) insufficiency is therefore neither the sole initiating mechanism nor a sufficient definition [1,2,3,4,5,6].

Genetic architecture contributes to this variability without creating a simple divide from sarcomeric cardiomyopathy. Nuclear mitochondrial disorders commonly follow autosomal or X-linked Mendelian inheritance. Transmitted mtDNA variants usually follow maternal inheritance, while many large-scale deletions arise sporadically; heteroplasmy and tissue-specific selection add further complexity. Sarcomeric cardiomyopathies also show age-dependent penetrance and variable expression, including among relatives carrying the same variant [1,2,7,8]. Genotype remains essential for diagnosis, but does not fully explain progression.

The myocardium depends on continuous ATP production and has small ATP stores relative to its turnover. This does not mean that cardiomyocytes lack spare respiratory capacity or metabolic flexibility. Healthy hearts increase oxidative ATP production and adjust substrate use as workload changes; inherited defects may restrict these responses to different degrees [9]. The mechanistic question is why some hearts remain compensated despite a mitochondrial lesion, whereas others develop progressive hypertrophy, fibrosis, or arrhythmia.

Cardiac involvement has important prognostic consequences. In a cohort of 223 genetically diagnosed pediatric patients with mitochondrial disease, cardiomyopathy was present in 21%, and left ventricular hypertrophy was associated with all-cause mortality after multivariable adjustment (hazard ratio 4.6, 95% confidence interval 2.8–7.3) [10]. An earlier cohort from the same program also reported poorer survival with cardiomyopathy; these related cohorts should not be treated as independent estimates of population risk [11]. In a neonatal-onset cohort, cardiomyopathy was the presenting phenotype in 38 of 281 patients [12]. These observations establish the clinical importance of cardiac disease without implying that a single cardiac feature predicts outcome across all mitochondrial genotypes.

Recognition can be delayed when mitochondrial disease resembles sarcomeric hypertrophic cardiomyopathy or when cardiac disease is overshadowed by neurological or skeletal-muscle manifestations [3,4]. Molecular testing that covers the relevant nuclear genes and mtDNA is central; tissue biochemical analysis can be useful when the molecular diagnosis remains unresolved [5]. Myocardial spectroscopy can characterize energetic impairment in selected cases, and rare pathological reports illustrate potentially severe conduction-system involvement [13,14]. These findings support combined structural, rhythm, and multisystem assessment rather than reliance on one biochemical or imaging result [6].

Therapeutic progress makes this mechanistic question clinically relevant. Elamipretide (Forzinity) received US accelerated approval in September 2025 to improve muscle strength in adults and children with Barth syndrome weighing at least 30 kg. Approval was based on knee-extensor muscle strength, an intermediate clinical endpoint, and requires confirmatory evidence of clinical benefit [15,16,17]. It does not establish prevention or reversal of cardiomyopathy. Gene replacement offers a different route, with cardiac rescue in animal models and preliminary human data on adeno-associated virus (AAV)-mediated frataxin (FXN) gene delivery (AAV-FXN), but important uncertainties concerning delivery, toxicity, durability, and cardiac outcomes remain [18,19,20,21,22].

We examine whether failure to match mitochondrial damage with effective quality control contributes to progression in selected inherited cardiomyopathies. Quality control is broader than mitophagy: it includes protein folding and degradation, mitochondrial dynamics, membrane maintenance, biogenesis, and disposal of damaged material [23,24,25]. We give mitophagy particular attention because it connects mitochondrial injury to lysosomal disposal and can be experimentally measured and perturbed. This is a testable rationale, not evidence that mitophagy is the dominant pathway or an obligatory intermediate in every genotype. Supportive and discordant disease-specific findings are assessed together in Section 4.4 [26,27,28,29,30,31,32,33].

**Figure 1:**
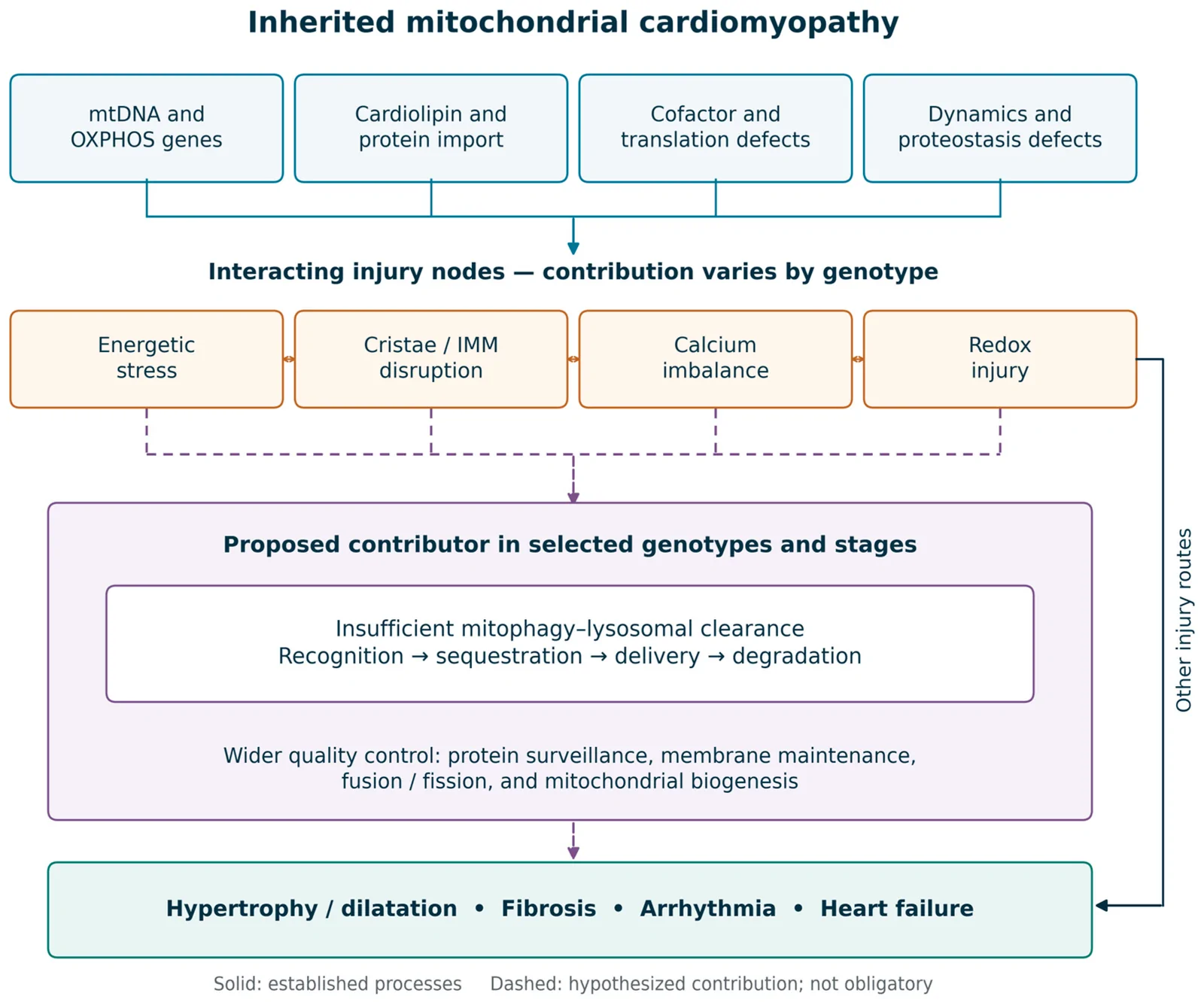
Evidence-informed framework for inherited mitochondrial cardiomyopathy. Primary genetic defects can perturb energetic supply, inner-membrane organization, calcium handling, and redox balance. The mitophagy–lysosome module is placed below these interacting injury nodes, within the wider mitochondrial quality-control system. Dashed arrows indicate the hypothesis that insufficient mitochondrial clearance amplifies injury in selected genotypes and stages; they do not imply an obligatory pathway in every disease. A separate route to cardiac injury is retained because energetic, electrical, and cell-death mechanisms may operate without demonstrable mitophagy impairment. The balance among damage, repair, clearance, and biogenesis determines the mitochondrial population. Disease-specific evidence and its limitations are discussed in Section 4.4. Blue boxes indicate genetic categories, orange boxes interacting injury processes, purple boxes the proposed clearance contribution within wider quality control, and the green box cardiac outcomes. Solid arrows indicate established relationships; double-headed arrows indicate interactions. Colors group concepts rather than encode quantitative data. mtDNA, mitochondrial DNA; OXPHOS, oxidative phosphorylation; IMM, inner mitochondrial membrane.

Our aim is to connect the genetic and functional diversity of mitochondrial cardiomyopathy with the strength of evidence for shared therapeutic targets. The review complements broad molecular and clinical accounts [1] and our previous discussion of the autophagy–lysosomal system in lysosomal storage cardiomyopathies [34]. Those disorders provide a conceptual comparison, not proof of an identical mechanism. Figure 1 distinguishes established disease mechanisms from the proposed contribution of impaired mitochondrial clearance, and Section 7 sets out predictions and alternative explanations.

### Literature Search and Selection

This is a critical narrative review, not a systematic review or meta-analysis. For the present revision, targeted PubMed/MEDLINE searches were performed through 12 September 2026, without a lower publication-date limit. Search combinations included “mitochondrial cardiomyopathy,” “mitochondrial quality control,” “mitophagy,” “lysosomal,” “tafazzin” or “Barth syndrome,” “frataxin” or “Friedreich ataxia,” “CPT2,” “FARS2,” “CHCHD10,” “PINK1,” “Parkin,” “BNIP3,” “NIX,” “FUNDC1,” “cardiolipin,” “elamipretide,” and “AAV.” Reference lists of relevant papers and reviews were examined, and selected full texts were checked through PubMed Central or publisher websites. US Food and Drug Administration (FDA) labeling and ClinicalTrials.gov records were consulted for approved indications and trial information. The search prioritized primary disease-specific cardiac studies, human therapeutic reports, and methodological studies that clarify what an assay measures. Noncardiac experiments and acquired-disease models were retained when mechanistically informative and identified as indirect evidence. Contradictory results and negative trials were retained. Published peer-reviewed reports were preferred to earlier preprints of the same work. Selection was based on relevance to the review questions; no exhaustive retrieval claim, formal risk-of-bias scoring, or quantitative pooling is made.

## 2. The Mitochondrion in the Cardiomyocyte

Four functional domains are relevant to mitochondrial cardiomyopathy: bioenergetic supply, inner-membrane architecture, calcium and redox handling, and the mechanisms that maintain or replace mitochondrial components. These domains interact, but their relative contribution differs by genotype, developmental stage, and physiological stress [1,9,23,24]. Their interaction provides a basis for convergence without requiring that every defect follow the same sequence.

### 2.1. Bioenergetic Demand and Substrate Flexibility

Mitochondria occupy roughly a third of cardiomyocyte volume. Fatty acid β-oxidation supplies much of the acetyl-coenzyme A (acetyl-CoA) used under normal adult conditions, while glucose, lactate, and ketone bodies contribute according to workload and availability [9]. ATP stores, spare respiratory capacity, and substrate flexibility are distinct: limited storage necessitates continuous synthesis, but does not imply that basal respiration already equals maximal oxidative capacity. The amount of reserve lost in an inherited disorder depends on residual enzyme function, substrate access, tissue context, and demand [1,9].

Experimental heart failure with preserved ejection fraction (HFpEF) and sarcomeric hypertrophic cardiomyopathy illustrate how fuel use can change in cardiac disease. An HFpEF model showed impaired insulin-stimulated glucose oxidation with predominant fatty acid oxidation, whereas empagliflozin improved metabolic coupling and cardiac function in a murine R403Q sarcomeric model [35,36]. These are indirect physiological comparisons; they do not establish the metabolic response or treatment benefit in a primary inherited mitochondrial cardiomyopathy.

### 2.2. The Inner Membrane: Cardiolipin, Cristae and Respiratory Organization

Cardiolipin contributes to inner mitochondrial membrane organization and interacts with respiratory complexes, metabolite carriers, and proteins involved in cristae maintenance [37,38]. Tafazzin remodels cardiolipin acyl chains. In cardiolipin remodeling-deficient human induced pluripotent stem cell (iPSC)-derived cardiomyocytes, maturation is accompanied by abnormal cristae and impaired respiratory development [38]. This supports a developmental contribution to disease but does not explain the full clinical timing of Barth syndrome, which can include fetal or neonatal cardiac manifestations [27,39]. Loss of mitoregulin also compromises membrane integrity in an ischemia–reperfusion model; that finding is informative about membrane biology but indirect for inherited cardiomyopathy [40].

Respiratory-chain complexes can associate in supercomplexes, but the functional consequences should not be stated as settled. Genetic manipulation has supported a role for supercomplex assembly in organizing electron flow, whereas experiments in mammalian heart mitochondrial membranes found no requirement for quinone channeling within supercomplexes [41,42]. Stabilization of individual complexes, membrane organization, and context-dependent effects on respiration remain plausible functions. A change in supercomplex abundance is therefore not, by itself, proof of improved ATP production or the mechanism of a clinical response.

### 2.3. Calcium Handling, Redox Balance and the Permeability Transition

Mitochondrial matrix calcium regulates dehydrogenases and helps couple contractile demand to substrate oxidation. Calcium loading and reactive oxygen species (ROS) can also promote opening of the mitochondrial permeability transition pore (mPTP). Experiments in isolated cardiac and hepatic mitochondria showed interactions between calcium and oxidant stress, while distinguishing pore opening from lipid-peroxidation-mediated membrane damage [43]. A rat fetal-hypoxia model further showed altered cyclophilin D abundance and calcium-retention capacity in adult offspring hearts [44]. These acquired and developmental stress models provide mechanistic context, not direct proof of the sequence operating in inherited disease. The contribution of permeability transition must be established in the relevant genotype rather than inferred from an elevated ROS marker alone.

### 2.4. Mitochondrial Dynamics and Proteostasis

Fusion, mediated by mitofusin 1 (MFN1) and mitofusin 2 (MFN2) at the outer membrane and optic atrophy protein 1 (OPA1) at the inner membrane, permits content mixing and supports mitochondrial function. Fission involves dynamin-related protein 1 (DRP1) (encoded by *DNM1L*) and recruitment factors including mitochondrial fission factor (MFF), and contributes to mitochondrial distribution and segregation of damaged regions [45]. These processes interact with protein surveillance. In mouse hearts, loss of the mitochondrial protease YME1-like ATPase 1 (YME1L) caused excessive OPA1 processing by OMA1 zinc metallopeptidase (OMA1) and cardiomyopathy; manipulating OMA1 or metabolism modified the phenotype [23]. Loss of mitochondrial Lon peptidase 1 (LONP1) in embryonic mouse cardiac tissue disrupted proteostasis and the developmental transition toward oxidative metabolism [24]. Neither finding requires mitophagy to be the sole mediator.

Coiled-coil-helix-coiled-coil-helix domain-containing protein 10 (CHCHD10) is an intermembrane-space protein involved in mitochondrial proteostasis and cristae maintenance, rather than a canonical fusion or fission enzyme. In knock-in mice, p.S55L, corresponding to human p.S59L, is associated with CHCHD10 aggregation, disrupted cytochrome c oxidation, and activation of mitochondrial stress signaling through OMA1, DAP3-binding cell death enhancer 1 (DELE1), and heme-regulated inhibitor kinase (HRI) [46]. Pregnancy precipitated postpartum heart failure in this model, and nicotinamide riboside with pterostilbene improved survival in the reported experiment [47]. These findings illustrate load-dependent vulnerability and a proteostasis/stress-response mechanism; they do not demonstrate that reduced mitophagy caused the cardiac phenotype.

### 2.5. Mitophagy and Lysosomal Completion

Mitophagy is the selective lysosomal degradation of mitochondria and is one component of mitochondrial quality control. Depolarization can stabilize PTEN-induced kinase 1 (PINK1) on the outer membrane and activate Parkin-dependent ubiquitination, facilitating autophagic recruitment. Receptor-mediated routes involving BCL2-interacting protein 3 (BNIP3), BCL2-interacting protein 3-like (BNIP3L, also called NIX), and FUN14 domain-containing protein 1 (FUNDC1) provide additional mechanisms of mitochondrial recognition [48,49,50]. Their activity depends on developmental stage, stress, and cell type. In vivo reporter studies demonstrate that basal mitophagy can persist without PINK1, and alternative mitophagy associated with Unc-51-like autophagy-activating kinase 1 (ULK1) and Ras-related protein Rab-9 (RAB9) contributes to cardiac adaptation during pressure overload [51,52,53]. PINK1/Parkin is therefore a well-characterized stress-responsive pathway, not an established universal controller of basal cardiac mitochondrial turnover.

Initiation, lysosomal delivery, and completed degradation are different steps. PINK1 or Parkin recruitment, microtubule-associated protein 1 light chain 3-II (LC3-II) or sequestosome 1 (p62) abundance, electron-microscopic vacuoles, and mitochondrial mass cannot individually determine the direction or rate of mitophagic flux [54]. Mitochondria-targeted Keima (mt-Keima) and tandem-fluorescence reporters such as mito-QC detect mitochondrial material in an acidic compartment and provide more direct evidence of lysosomal delivery [52,55]. At a single time point, however, this signal reflects accumulated delivery and persistence rather than a complete degradation rate. Interpretation requires time-course measurements, assessment of lysosomal pH and proteolysis, and complementary turnover assays or appropriately controlled lysosomal inhibition. General autophagic flux also should not be equated with mitochondria-specific flux. The broader quality-control processes are summarized in Table 1; disease-specific assay findings are discussed in Section 4.4.

**Figure 2:**
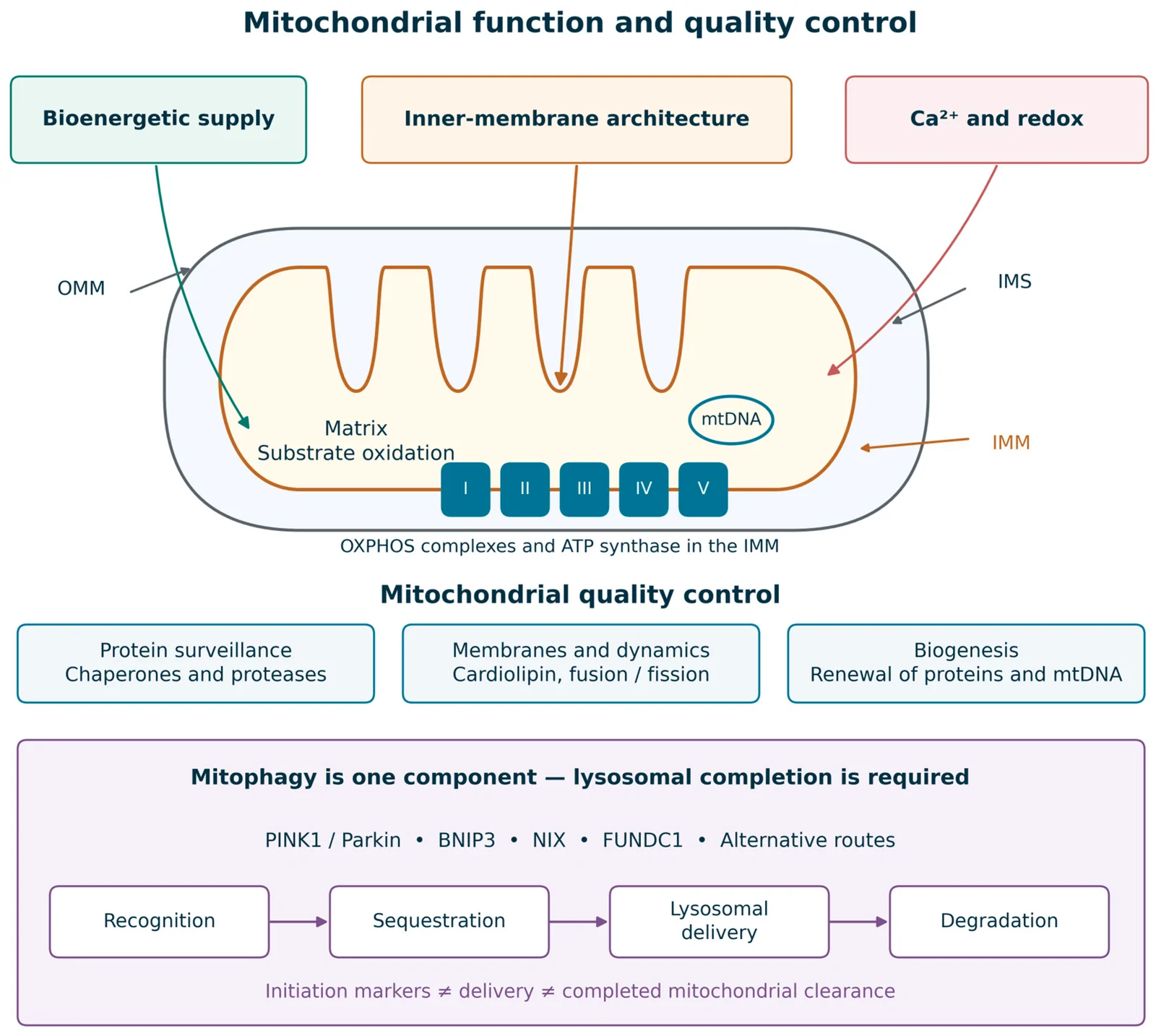
Mitochondrial compartments and the wider quality-control system in cardiomyocytes. Inner-membrane architecture is shown by the continuous crista-forming membrane. Respiratory complexes and ATP synthase are located in the inner membrane; the matrix contains mtDNA and metabolic machinery. Protein surveillance, fusion/fission, membrane maintenance, and biogenesis contribute to mitochondrial quality control. Mitophagy proceeds through recognition, sequestration, lysosomal delivery, and degradation, with PINK1/Parkin-dependent and receptor-mediated inputs. The schematic is not to scale and does not imply that respiratory supercomplex formation necessarily increases catalytic efficiency. OMM, outer mitochondrial membrane; IMM, inner mitochondrial membrane; IMS, intermembrane space. mtDNA, mitochondrial DNA; OXPHOS, oxidative phosphorylation; ATP, adenosine triphosphate; Ca^2+^, calcium ions; PINK1, PTEN-induced kinase 1; BNIP3, BCL2-interacting protein 3; NIX, BCL2-interacting protein 3-like; FUNDC1, FUN14 domain-containing protein 1. The colored boxes distinguish functional domains, wider quality control, and mitophagy. Arrows identify compartments or indicate the sequence of mitophagy steps; colors do not represent measured values.

The key distinction is between mitochondrial injury and the response to that injury. Structural damage, redox stress, and calcium imbalance can interact, but clearance may increase, decrease, or become insufficient relative to damage [28,29,30,31,32,33,38,43]. Figure 2 places these functions within the mitochondrial compartments and separates the wider quality-control system from the mitophagy–lysosome pathway. The lysosomal interface also explains the conceptual overlap with storage-disease cardiomyopathies, while leaving the disease-specific causal relationships to be tested [34].

## 3. Genetic Architecture of Inherited Mitochondrial Cardiomyopathy

Inherited mitochondrial cardiomyopathy is a two-genome problem with variable tissue expression. Nuclear variants follow Mendelian inheritance and affect respiratory-chain structure and assembly, mtDNA maintenance, protein import, cardiolipin remodeling, iron–sulfur cluster biogenesis, and mitochondrial dynamics or proteostasis [1,2]. mtDNA variants add heteroplasmy, replicative segregation, and tissue-specific selection; maternal transmission applies to inherited mtDNA variants, whereas single large-scale deletions are often sporadic [5,7]. A nuclear maintenance defect can produce secondary mtDNA depletion or deletions, and an mtDNA translation defect can affect nuclear-encoded respiratory complexes. These categories overlap biologically and do not predict the mitophagy phenotype on their own.

### 3.1. mtDNA Point Variants and Large-Scale Rearrangements

Pathogenic mtDNA variants associated with cardiomyopathy occur in protein-coding and transfer-RNA genes. For example, m.3243A>G in *MT-TL1* may accompany multisystem disease, whereas m.4300A>G in *MT-TI* can cause cardiac-predominant disease or cardiomyopathy with extracardiac manifestations [1,13]. Low or declining blood heteroplasmy does not exclude clinically important disease. Urinary epithelial cells or skeletal muscle may improve detection of certain variants, including m.3243A>G, depending on the variant, age, and tissue distribution [5,57]. This is a diagnostic advantage, not evidence that heteroplasmy in those tissues estimates the myocardial mutant load. Myocardial heteroplasmy is spatially heterogeneous and generally unavailable without invasive sampling; tissue choice should follow the diagnostic question, and cardiac risk assessment should rely on clinical evaluation, imaging, and rhythm surveillance.

Single large-scale mtDNA deletions can cause the Pearson–Kearns–Sayre spectrum. Progressive atrioventricular block and His–Purkinje conduction disease may precede overt ventricular dysfunction and can lead to sudden death [7]. A preserved ejection fraction therefore does not exclude clinically important cardiac involvement. Serial electrocardiography and rhythm surveillance should accompany structural assessment, with specialist evaluation when conduction abnormalities emerge [6].

### 3.2. Nuclear OXPHOS, Assembly and mtDNA-Maintenance Genes

Nuclear-encoded disease expands the phenotype beyond classical maternal inheritance. Biallelic variants affecting structural respiratory-chain subunits or assembly factors—including *NDUFS2*, *NDUFV2*, *NDUFS4*, *ACAD9*, *SCO2*, *COX10*, *COX15*, *BCS1L* and *TMEM70*—may present with neonatal or infantile hypertrophic cardiomyopathy, dilated cardiomyopathy, noncompaction, lactic acidosis or rapidly progressive multisystem failure [1,2]. The clinical course is determined not simply by which complex is affected, but by residual activity, tissue distribution, and whether the defect is amenable to cofactor rescue. *ACAD9* deficiency is the clearest example: riboflavin improves complex I activity or clinical status in a substantial, but not universal, subset, making molecular diagnosis directly actionable [58].

Genes responsible for mtDNA replication and maintenance, including *POLG*, *TWNK*, *TK2*, and *MPV17*, can produce secondary depletion or multiple deletions. Cardiac involvement varies by gene and phenotype and may be overshadowed by hepatic, neurological, or skeletal-muscle disease [1,2]. Follow-up should be matched to the established gene–disease relationship and individual manifestations, rather than assuming that all maintenance disorders carry the same cardiac risk [6].

### 3.3. Cardiolipin, Cofactor and Protein-Import Disorders

Barth syndrome results from X-linked loss-of-function variants in *TAFAZZIN* (historically *TAZ*), which impair cardiolipin remodeling. Increased monolysocardiolipin relative to mature cardiolipin is associated with abnormal mitochondrial membrane organization and development; the cardiac phenotype can include dilatation, hypertrophy, noncompaction, and arrhythmia [27,38,39]. Friedreich ataxia usually results from biallelic GAA-repeat expansions in *FXN*. Frataxin deficiency disrupts iron–sulfur cluster biogenesis and iron handling; hypertrophic remodeling may progress to fibrosis, systolic dysfunction, or arrhythmia [20,30,31,59]. Sengers syndrome results from biallelic variants in the acylglycerol kinase gene (*AGK*). AGK functions as a lipid kinase and a component of the translocase of the inner mitochondrial membrane 22 (TIM22) carrier-import complex, linking lipid metabolism with protein import and the clinical combination of cataracts, lactic acidosis, and cardiomyopathy [60,61]. These primary lesions justify a broad definition of mitochondrial disease, but do not establish an identical downstream clearance defect.

### 3.4. Dynamics Genes, Inheritance and Sex

Pathogenic variants affecting mitochondrial dynamics often cause neurological or multisystem disease, and the strength of evidence for cardiac involvement differs among genes. *OPA1*, *MFN2*, and *DNM1L* should therefore not be presented as interchangeable causes of isolated cardiomyopathy [1,2,45]. Experimental protease and dynamics perturbations demonstrate that network remodeling can modify cardiac disease, while CHCHD10 illustrates a related proteostasis defect [23,24,46]. For each proposed model, disease relevance should be distinguished from its usefulness as a pathway perturbation.

Inheritance also changes ascertainment. X-linked *TAZ* disease predominantly affects males, whereas heteroplasmic mtDNA disease can show sex-dependent penetrance despite maternal transmission. Experimental work has begun to identify biological rather than purely ascertainment-based explanations. In a mouse model of mitochondrial cardiomyopathy, spatial and single-cell transcriptomics identified a transition regulated by activating transcription factor 3 (ATF3) with sex-specific features, suggesting that stress-response state, hormonal context, and cellular composition modify progression after the initiating energetic lesion [62]. These observations are not yet a basis for sex-specific treatment, but they argue that sex should be prespecified in mechanistic studies and stratified in natural-history cohorts.

## 4. Interacting Mechanisms of Cardiac Injury

Energetic stress, altered redox and calcium handling, inner-membrane disruption, and mitochondrial turnover can interact in inherited cardiomyopathy. However, neither their order nor their relative importance is uniform across genotypes [1,28,29,30,31,32,33,46]. Inadequate clearance may amplify injury in some settings, while in others mitophagy is preserved or increased. The following sections separate plausible interactions from evidence for a necessary causal step.

### 4.1. Bioenergetic Failure and Maladaptive Remodeling

Reduced ATP-generating capacity may initially be buffered by altered substrate use, mitochondrial biogenesis, and stress-response signaling [9,30]. Mitochondrial proliferation can increase cellular mass without proportionate restoration of respiratory function. Adenosine monophosphate-activated protein kinase, mechanistic target of rapamycin (mTOR), and the integrated stress response connect metabolic status with growth and protein synthesis. Disease models show that these responses can be adaptive or harmful: frataxin-deficient hearts exhibit substantial changes in biogenesis and oxidized nicotinamide adenine dinucleotide (NAD+) metabolism, and limiting OMA1-dependent stress signaling modifies CHCHD10-associated cardiomyopathy despite persistent mitochondrial abnormalities [30,46]. Hypertrophy should therefore be viewed as a composite response, not solely the consequence of ATP depletion or impaired mitophagy.

### 4.2. ROS, Calcium and mPTP Opening

ROS can damage respiratory proteins and membrane lipids, while impaired ATP supply can compromise calcium reuptake and ionic homeostasis. Interactions between calcium and oxidant stress can promote mitochondrial permeability transition, providing a route to membrane-potential loss and cell injury [43]. The extent of oxidative stress nevertheless varies among inherited models: a recent cardiac frataxin-deficiency study reported mitochondrial iron accumulation and impaired respiration without increased measured oxidative stress [31]. ROS production, pore opening, and mitochondrial clearance should consequently be measured as distinct processes. Injury through calcium or cell-death pathways does not, by itself, demonstrate mitophagy failure.

### 4.3. Cristae Disruption and Respiratory Disorganization

Cardiolipin remodeling defects, altered OPA1 processing, and proteostasis disturbances can disrupt cristae and respiratory organization [23,38,46]. These changes may influence respiration, cytochrome c behavior, and vulnerability to injury, but the magnitude and mechanism are model-dependent. Supercomplex abundance and cristae morphology should be interpreted alongside functional measurements rather than used as proxies for catalytic efficiency [41,42]. This distinction is also important therapeutically: biological effects on the inner membrane do not establish a cardiac clinical benefit from a cardiolipin-directed drug.

### 4.4. Mitophagy as a Context-Dependent Contributor

Barth syndrome provides disease-specific support, with important assay limitations. Tafazzin-deficient fibroblasts showed impaired mitophagosome initiation, and isogenic human cardiomyocytes showed a blunted depolarization-induced mitochondrial-to-lysosomal delivery response [26,27]. In *Taz*-knockout mice, cardiomyopathy and dye-based mitophagy readouts differed by genetic background despite similar cardiolipin abnormalities; delivery-associated signals decreased in a susceptible background but increased in a more permissive background [28]. Rapamycin improved cardiac function in a *Taz*-knockdown model, accompanied by changes in autophagic and lysosomal markers [29]. This intervention supports the relevance of the pathway, but its pleiotropic mTOR effects and indirect cardiac clearance readouts do not isolate mitophagy as the sole mediator.

Frataxin deficiency does not produce a uniformly suppressed response. A muscle creatine kinase conditional *Fxn*-knockout study used bafilomycin-sensitive accumulation of autophagic proteins and MFN1 to support increased turnover in mouse hearts; the human myocardial observations were marker-based [30]. A later cardiac-specific model implicated lysosomal dysfunction using p62/Parkin and findings related to mTOR and transcription factor EB (TFEB) [31]. In *Drosophila* glia, frataxin depletion increased a mitochondria-targeted Rosella (mtRosella) delivery-associated signal, providing an indirect, noncardiac counterexample to uniformly suppressed mitophagy [32]. Differences in genotype construction, tissue, age, stage, and assay may explain some discordance, but a single temporal sequence has not been established. These data argue against treating reduced mitophagy as a necessary consequence of frataxin deficiency.

Deficiency of mitochondrial phenylalanyl-tRNA synthetase 2 (FARS2) adds evidence from a mitochondrial translation defect. The reported cardiac models showed stage-related changes in autophagy-associated markers, while bafilomycin and tandem LC3 experiments in neonatal cardiomyocytes supported increased general autophagic flux [33]. These assays do not establish selective mitochondrial degradation throughout the disease course. Rescue after modifying fission or fusion also indicates that broader network homeostasis may be relevant, rather than implying that increasing mitophagy is always beneficial.

The carnitine palmitoyltransferase 2 (CPT2)-deficient mouse heart provides a useful mechanistic experiment in fatty acid oxidation deficiency. mt-Keima indicated reduced lysosomal delivery, and deletion of *Usp30*, which encodes ubiquitin-specific peptidase 30, improved this readout, cardiac function, and survival [56]. This is direct evidence within that metabolic model but indirect support for extension to primary respiratory-chain, cardiolipin, or mtDNA disorders. HFpEF experiments similarly show that mitochondrial dysfunction need not evoke an adequate mitophagic response, but remain acquired-disease evidence [63]. Neither establishes cross-genotype therapeutic efficacy.

The working hypothesis is therefore that inadequate mitochondrial clearance contributes to progression in some genotypes and stages, not that every mitochondrial cardiomyopathy requires defective mitophagy. Excessive removal can also be harmful if biogenesis and residual respiratory capacity cannot maintain a functional mitochondrial population [33,54]. “Restoration” should mean correction of a demonstrated turnover defect, which may require improved lysosomal completion or a different balance of repair and removal, rather than indiscriminate induction. Table 2 summarizes the disease- and gene-specific evidence and its assay limitations.

### 4.5. From Mitochondrial Injury to Hypertrophy, Fibrosis and Arrhythmia

The cardiac phenotype reflects interactions among cardiomyocytes, fibroblasts, vascular cells, and the conduction system. Energetic and calcium disturbances may alter ion transport and contractility, while cell loss and fibrotic replacement can create a further arrhythmic substrate [1,43,59]. These routes can coexist with altered mitochondrial turnover but are not proof that it is upstream. Large-scale mtDNA deletions may be conduction-predominant, Barth syndrome combines myocardial disease with arrhythmic risk, and Friedreich ataxia can develop fibrosis before severe systolic decline [7,39,59]. The extent to which correcting mitochondrial dysfunction reverses established scar remains uncertain.

## 5. Biomarkers: From Detection to Mechanistic Stratification

No circulating marker currently identifies mitochondrial cardiomyopathy with adequate cardiac specificity. Biomarkers are nevertheless useful when assigned a precise job: screening for systemic mitochondrial stress, quantifying myocardial phenotype, selecting a mechanistic subgroup, or measuring pharmacodynamic response. Problems arise when one marker is expected to perform all four tasks [6,64,65,66,67].

Lactate is affected by collection technique, exertion, perfusion, hepatic clearance, and acute illness; normal values do not exclude mitochondrial disease [5]. Growth differentiation factor 15 (GDF-15) and fibroblast growth factor 21 (FGF-21) can support recognition of systemic mitochondrial stress. Their diagnostic performance varies across cohorts, and FGF-21 is particularly associated with mitochondrial translation or maintenance disorders involving skeletal muscle [64,65,67]. GDF-15 also rises with aging, inflammation, malignancy, renal dysfunction, and conventional heart failure. Neither marker localizes disease to myocardium, measures cardiac mitophagy, or is validated as a surrogate endpoint for mitochondrial cardiomyopathy progression.

Circulating cell-free mtDNA is an exploratory marker of mitochondrial release and tissue injury, with uncertain cellular origin and substantial preanalytical sensitivity. The cited Fabry-disease report concerns a lysosomal storage disorder and therefore provides indirect evidence, not validation in primary inherited mitochondrial cardiomyopathy [66]. Plasma cell-free mtDNA should not be equated with myocardial mutant load or myocardial mitophagic flux, and its use as a cardiac treatment-response marker requires prospective validation.

Cardiac assessment supplies the organ-level information that systemic biomarkers lack. Echocardiography evaluates wall thickness, chamber size, systolic and diastolic function, and, when feasible, strain. Cardiac magnetic resonance (CMR) adds ventricular volumes, mass, and tissue characterization, including late gadolinium enhancement (LGE) and T1/extracellular-volume mapping [1,6]. Phosphorus-31 magnetic resonance spectroscopy (31P-MRS) can measure the phosphocreatine-to-ATP ratio, an index of myocardial energetic state; it does not directly measure maximal respiratory reserve or mitophagic flux [13]. Imaging should be paired with rhythm surveillance, and CMR fibrosis measures should not be treated as a validated threshold of treatment irreversibility. Table 3 summarizes these measures and their limitations.

A trial may combine genotype, cardiac imaging, rhythm burden, conventional cardiac biomarkers, and exploratory systemic measures, with each assigned a prespecified role. Improvement in GDF-15, FGF-21, or exercise performance may arise from skeletal muscle or other organs and should not be interpreted as cardiac rescue without concordant cardiac outcomes [6,65,67]. Whether a composite signature improves prediction beyond genotype and conventional cardiac variables remains a research question.

## 6. Targeted Therapy

Supportive heart-failure, conduction, and arrhythmia management remains essential and should be integrated with multisystem care [6]. Mechanism-directed strategies are considered below by target, with cellular and animal findings separated from human trials and approved indications. Evidence of mitochondrial target engagement, improvement in cardiac function, and reduction in clinical events are distinct outcomes; success at one level does not establish the next.

### 6.1. Cardiolipin-Directed Therapy: Elamipretide/Forzinity

Cellular and animal evidence. Elamipretide is a mitochondria-targeting tetrapeptide that binds cardiolipin and localizes to the inner membrane [17,37]. It does not replace tafazzin or correct the causal variant. Studies in tafazzin-deficient mice reported improvements in mitochondrial morphology and mitophagy-associated proteins, but these measurements do not demonstrate that selective restoration of completed mitochondrial degradation mediates the effect [68].

Human trials. In the 12-participant randomized crossover phase of TAZPOWER, elamipretide did not outperform placebo on the two primary endpoints, six-minute walk distance and the Barth Syndrome Symptom Assessment total fatigue score [17,69]. The 168-week open-label extension reported functional and selected echocardiographic improvements, but its small sample, attrition, and lack of a concurrent long-term control limit causal inference [70]. These observations are hypothesis-generating for cardiac efficacy.

Approved indication. The FDA granted accelerated approval in September 2025 for improvement of muscle strength in adults and pediatric patients with Barth syndrome weighing at least 30 kg. The supporting intermediate clinical endpoint was knee-extensor muscle strength; continued approval is contingent on confirmatory evidence of clinical benefit [15,16,17]. The product is not approved as a treatment for mitochondrial cardiomyopathy as a class, and reduction in cardiac events or cardiac disease modification has not been established. This indication also does not establish treatment benefit for the infants in whom Barth cardiomyopathy can be most severe.

### 6.2. Gene Replacement and Mitochondrial Genome Editing

Animal gene-replacement evidence. Nuclear-encoded defects can be addressed by transgenes whose products are imported into mitochondria. AAV-mediated *TAFAZZIN* replacement improved mitochondrial and cardioskeletal function in mouse Barth models, including prevention and reversal of heart failure [18,19]. AAV-mediated *FXN* expression also prevented or reversed severe cardiomyopathy in a frataxin-deficient mouse model [20]. These are genotype-specific preclinical results and do not establish efficacy in human Barth syndrome or across mitochondrial diseases.

Early human AAV-FXN evidence. A 2026 pooled report of two nonrandomized, open-label dose-escalation trials included 17 adults with Friedreich ataxia cardiomyopathy [22]. Cardiac frataxin increased in all eight participants with a three-month biopsy; exploratory CMR left ventricular mass index decreased by at least 10% in nine participants and was stable in eight. Four serious adverse events were reported: three possibly related to prednisone immunosuppression and one possibly vector-related myocarditis; all resolved. These findings support target engagement and further study, but the absence of a randomized comparator and the small sample preclude establishing cardiac efficacy or long-term safety. The associated registry record, including NCT05445323, should be distinguished from the published clinical report [21,22].

Safety considerations shared by AAV strategies. Systemic vector delivery can cause immune-mediated toxicity, liver injury, complement-associated thrombotic microangiopathy, and tissue inflammation. These are relevant development risks for both AAV-TAZ and AAV-FXN, rather than an FXN-specific limitation [71]. The observed rate depends on vector, dose, transgene, patient factors, and immunosuppression; it should not be assumed identical across products. FXN additionally has a transgene-expression risk: excessive frataxin caused cardiac or hepatic toxicity in mouse studies [72,73]. Pre-existing immunity, durability in growing children, uneven tissue distribution, and limited redosing remain constraints. The risks of immunosuppression also need to be considered in patients with multisystem disease.

Cellular and animal mtDNA-editing evidence. Protein-only DddA-derived cytosine base editors (DdCBEs) and transcription activator-like effector-linked adenine editors avoid the guide-RNA delivery problem that limits conventional clustered regularly interspaced short palindromic repeats (CRISPR)-Cas editing in mitochondria [74,75,76]. Targeted editing has been demonstrated in cultured cells and, for DdCBE delivery, in postmitotic mouse tissues including heart [74]. These studies include installation of sequence changes and disease modeling; they should not be collectively described as therapeutic correction. Translation requires sufficient editing of relevant cardiomyocytes, suitable sequence scope, control of bystander and off-target changes, and safe delivery. There is no established clinical cardiac benefit from mtDNA base editing.

### 6.3. Cofactor, Substrate-Bypass and Mitophagy-Directed Approaches

Human cofactor evidence. Riboflavin can improve biochemical or clinical findings in a subset of patients with ACAD9 deficiency, although responses are not uniform and the available clinical evidence is largely observational [58]. Coenzyme Q (CoQ) supplementation is most rational for defined primary CoQ deficiencies; results should not be generalized to all OXPHOS disorders [5]. In Friedreich ataxia, early idebenone studies suggested reduced hypertrophy, whereas a larger randomized study did not show improvement in cardiac structure or function over six months [77,78]. Mechanistic plausibility does not substitute for controlled cardiac outcomes.

Preclinical substrate and redox approaches. NAD+ replenishment and substrate manipulation may help in selected contexts, as illustrated by the CHCHD10 mouse intervention, but the appropriate strategy depends on the blocked pathway and potential accumulation of toxic intermediates [30,47]. Respirometry, isotope tracing, and energetic measurements can assess metabolic effects in models. Improved exercise performance alone would not demonstrate myocardial benefit in a multisystem disorder.

Preclinical mitophagy-directed interventions. *Usp30* deletion in CPT2-deficient hearts provides genetic proof of concept within a fatty acid oxidation model, not evidence that a USP30 drug treats inherited mitochondrial cardiomyopathy [56]. The Barth rapamycin study provides a separate disease-specific intervention, but mTOR inhibition affects growth, metabolism, general autophagy, and lysosomal biology [29]. FARS2 findings further caution that reducing excessive fission or autophagic activity can be beneficial in a specific experimental context [33]. No selective mitophagy-modulating treatment has established clinical efficacy for these cardiomyopathies. Development should require a demonstrated turnover abnormality, evidence that the intended step is corrected, preserved mitochondrial mass and respiration, and improvement in cardiac outcomes; inhibition or enhancement should follow the measured defect.

### 6.4. Therapeutic Choice in Multisystem Disease

For Barth syndrome, elamipretide has the nearer-term clinical advantage of an approved muscle-strength indication in the eligible population. AAV-TAZ has the stronger etiologic rationale because it seeks to restore the missing remodeling enzyme, and its mouse cardiac results justify clinical development [17,18,19]. Current evidence cannot rank their ability to prevent heart failure: there is no head-to-head comparison, and their maturity, exposure, reversibility, and risks differ. Complementary or sequential use is conceivable, but has not been established. A useful comparison would require cardiac target engagement, ventricular function and rhythm outcomes, durability, and systemic safety, rather than relying only on cardiolipin-related biomarkers.

Multisystem disease may determine both treatment feasibility and apparent response. In Barth syndrome, skeletal myopathy, growth impairment, and neutropenia can influence exercise endpoints, dose eligibility, infection risk, and tolerance of immunosuppression [17,39]. In Friedreich ataxia, neurological disability, skeletal-muscle involvement, and diabetes can limit exercise independently of cardiac function; cardiac gene delivery does not imply equivalent neurological benefit [6,22,59]. Trials should assess cardiac and extracardiac outcomes separately, document organ function relevant to toxicity, and incorporate patient priorities. A heart-directed treatment may still be valuable when neurological disease persists, but its expected benefit and limitations must be explicit. Table 4 summarizes the therapeutic evidence and remaining cardiac questions.

## 7. A Falsifiable Framework

We propose that, in a subset of inherited mitochondrial cardiomyopathies, an imbalance between mitochondrial damage and effective lysosomal clearance contributes to progression beyond the initiating genetic lesion. This hypothesis does not make impaired mitophagy necessary or sufficient for cardiomyopathy, and does not equate mitophagy with the entire quality-control system. Comparable disease may arise through persistent respiratory failure, altered proteostasis or stress signaling, calcium instability, inflammation, or defects in mitochondrial renewal [23,24,28,29,30,31,32,33,46]. The contribution of each mechanism must be tested rather than assumed.

Prediction 1 concerns progression. Among models with comparable respiratory impairment, a reproducible deficit in mitochondria-specific turnover should precede or predict worsening cardiac injury in the subgroup to which the hypothesis applies. Preserved or increased completed clearance despite progression would argue against the proposed bottleneck in that setting. A higher damage burden cannot be inferred solely from low flux; the two must be measured independently.

Prediction 2 concerns intervention. Selective correction of an identified clearance defect, without correction of the causal variant, should improve cardiac function while maintaining adequate mitochondrial mass and respiratory capacity. Rescue should be attenuated by independent disruption of the relevant clearance pathway. Functional benefit without a turnover change, or no benefit despite verified correction of turnover and appropriate timing, would favor an alternative mechanism. Because most candidate drugs are pleiotropic, association between a reporter signal and rescue is not sufficient.

**Figure 3:**
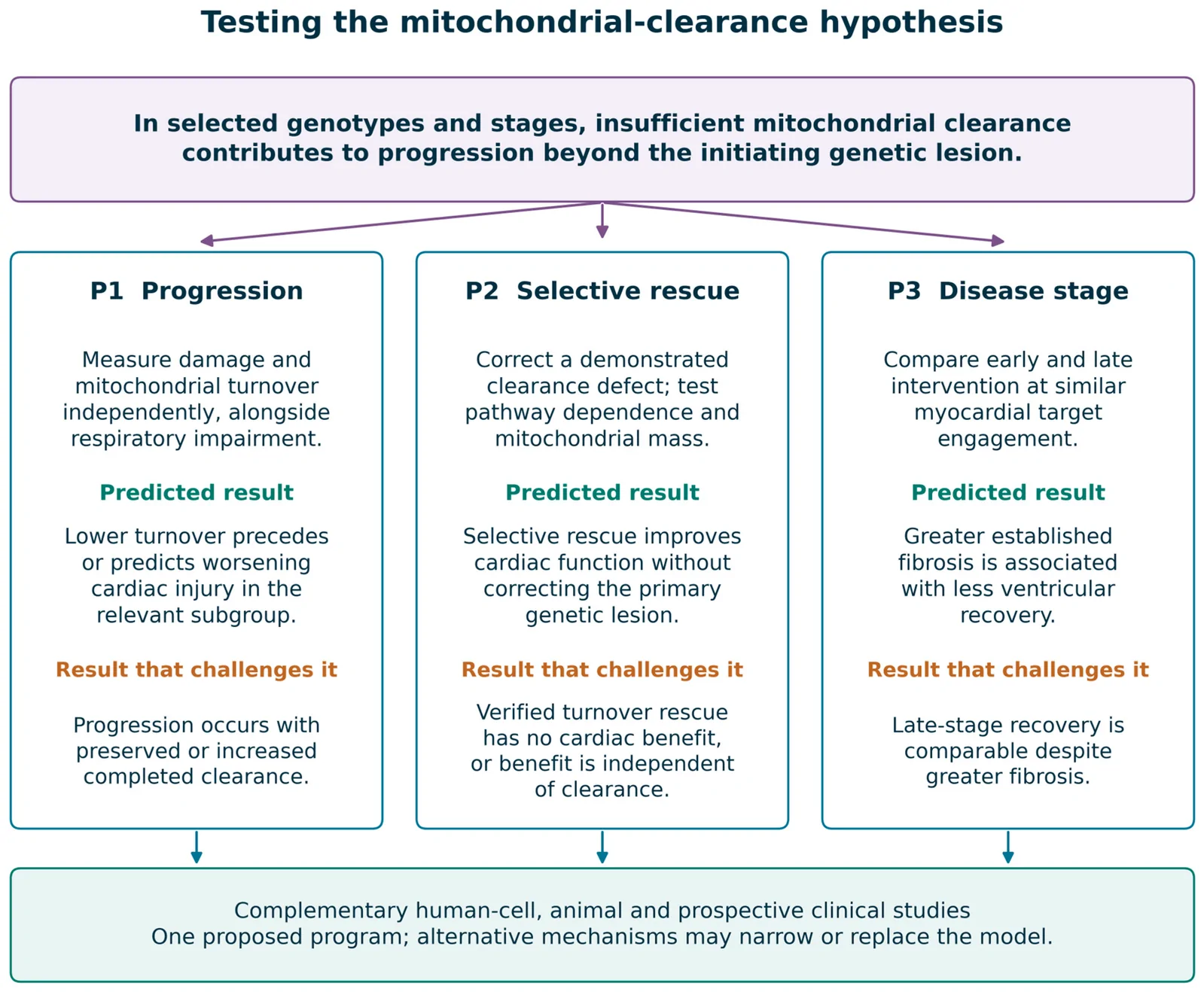
Central hypothesis, three predictions, and informative negative results. The framework tests whether deficient mitochondrial clearance contributes to progression in a defined genotype and disease stage. Prediction 1 tests temporal association beyond respiratory impairment; Prediction 2 tests selective rescue and pathway dependence; Prediction 3 tests whether established fibrosis modifies recovery despite comparable target engagement. Negative findings narrow or weaken the relevant component rather than being reclassified automatically as an unmeasured quality-control defect. Human cell models, genotype-relevant animals, and prospective clinical studies provide complementary evidence. This is one proposed research program, not an exhaustive or mandatory sequence.

Prediction 3 concerns disease stage. With increasing established fibrosis, similar mitochondrial target engagement may yield less ventricular recovery. This is a proposed effect modifier, not a known fibrotic ceiling or a validated CMR cutoff. Fibrosis should initially be analyzed as a continuous variable alongside viable myocardium, disease duration, and arrhythmic substrate. Equivalent late recovery would challenge the timing prediction; failure to recover without adequate myocardial target engagement would not test it. Figure 3 links the three predictions to measurements and outcomes that would weaken them.

### 7.1. Stage 1: Complementary Cellular and Animal Mechanistic Studies

Stage 1 should use complementary human and animal systems rather than an iPSC-only gate. Isogenic iPSC-derived cardiomyocytes, engineered cardiac tissues, isolated adult cardiomyocytes, and genotype-relevant animals with mitochondrial reporters can be studied in parallel [27,28,33,52,55]. Genotype selection should span mechanistic classes: *TAFAZZIN* for lipid remodeling, *FXN* for iron–sulfur biology, an assembly defect such as *ACAD9*, *AGK* for import, *FARS2* for translation, an mtDNA variant, and a dynamics/proteostasis model such as *CHCHD10*. These are illustrative candidates, not a requirement that every model be included or that every gene have equivalent clinical or experimental validity.

Basal and stress-induced measurements should combine mitochondrial delivery reporters with lysosomal pH, proteolytic capacity, time-resolved mitochondrial-substrate turnover, biogenesis, and respiratory mass [52,54,55]. Respiratory reserve, ATP/adenosine diphosphate (ADP), membrane potential, ROS, calcium handling, and contractile or electrophysiological function should be measured separately. Genetic correction, matched heteroplasmy controls where feasible, and pathway-specific perturbations of PINK1/Parkin, receptor-mediated routes, lysosomal completion, and proteostasis can distinguish competing mechanisms. Negative results are informative only when reporter behavior, maturation, lysosomal competence, and target engagement are verified.

### 7.2. Stage 2: In Vivo Intervention and Timing

Stage 2 can test early and later intervention in relevant animal models using gene replacement, membrane-directed treatment, and a mechanistically justified turnover intervention. Longitudinal cardiac imaging and telemetry should be paired with tissue-specific delivery/turnover assays, mitochondrial mass, respiration, histology, and extracardiac safety measurements [19,20,29,33,52,55,56]. Factorial designs and independent pathway perturbations can test whether clearance is required for benefit or merely accompanies it. Fibrosis, exposure, disease duration, and viable myocardium should be modeled as potential modifiers, avoiding a predefined irreversible threshold. Human cell results and animal findings should inform each other throughout, rather than one system being treated as conclusive.

### 7.3. Stage 3: Human Correlative and Clinical Evaluation

A prospective, genotype-stratified registry can relate imaging, rhythm burden, conventional cardiac biomarkers, clinical events, and systemic disease to longitudinal outcome. Patient-derived cells may provide complementary experimental phenotypes, but are not validated surrogates for myocardial turnover. No clinically established noninvasive test directly measures completed cardiac mitophagy; GDF-15, FGF-21, circulating mtDNA, and myocardial energetics should not be substituted for it [6,13,54,64,65,66,67]. An exploratory cellular or composite signature should undergo prespecified evaluation and external validation beyond genotype, age, and baseline cardiac status. Failure of an indirect signature would challenge its predictive usefulness, not by itself disprove a myocardial mechanism.

For interventional studies, cardiac target engagement and safety should precede claims of efficacy. Randomized designs are preferable when feasible; carefully matched natural-history controls can assist interpretation in ultrarare disease but cannot remove confounding. Short-term imaging or biomarker changes should remain distinct from prevention of heart-failure hospitalization, malignant arrhythmia, or death. Multisystem and patient-reported outcomes should be measured alongside cardiac endpoints, because benefit in one organ may coexist with ongoing disease elsewhere [6,22,69,70].

These three stages are a proposed framework, not an exhaustive list or a required sequence. Additional approaches include cardiac organoids, multicellular systems with fibroblasts and immune cells, spatial or single-cell analyses, direct study of protein import and proteostasis, metabolite tracing, and analysis of mitochondria-derived vesicles or organelle extrusion [23,24,25,27,46,62]. Such studies may identify pathways that explain progression or treatment response better than mitophagy. The framework should be narrowed or revised when those alternatives fit the evidence more closely.

## 8. Conclusions and Outlook

Inherited mitochondrial cardiomyopathies share several forms of cellular stress, but current evidence does not establish defective mitophagy as a universal mechanistic hinge. Mitochondrial quality control offers a useful broader framework because it includes repair, proteostasis, network remodeling, renewal, and disposal. Within that framework, impaired mitochondrial clearance is a plausible contributor in selected diseases and stages; supportive Barth syndrome experiments and discordant frataxin and FARS2 findings define both the rationale and its limits [26,27,28,29,30,31,32,33].

The near-term priority is to determine which patients and models actually have a clearance defect, where it occurs, and whether correcting it changes cardiac outcome. This requires harmonized assays that distinguish lysosomal delivery from degradation, independent measurement of mitochondrial damage and biogenesis, replication across genotypes, and interventions that discriminate mitophagy from broader metabolic effects [52,54,55]. An equally useful result would be the identification of subgroups in which proteostasis, calcium handling, or another pathway is more important; those findings should redirect treatment development rather than be forced into a common model.

Clinical translation should maintain the same distinctions. Elamipretide provides an approved muscle-strength indication in eligible patients with Barth syndrome, not established cardiac disease modification [17]. Early human AAV-FXN observations justify further controlled evaluation, while vector safety, durability, and multisystem benefit remain open questions [22,71,72,73]. The next studies should connect genotype and target engagement with meaningful cardiac and extracardiac outcomes, and test treatment timing without assuming a fixed fibrosis threshold. A mechanism-guided approach will be useful only if it permits the proposed role of mitophagy to be confirmed, restricted, or rejected in the populations studied.

## Figures and Tables

**Table 1:** Components of mitochondrial quality control and their relationship to cardiac homeostasis.

Process	Representative Effectors	Role	Interpretive Boundary	Ref.
Protein surveillance	LONP1; YME1L; OMA1; chaperones	Protein folding, proteolysis, and stress-response regulation	Protease and stress-response effects need not be mediated by mitophagy.	[23,24,25,46]
Membrane maintenance	tafazzin; OPA1; mitoregulin	Cardiolipin remodeling and cristae organization	Membrane or supercomplex changes are not themselves turnover measurements.	[38,40,41,42]
Fusion and fission	MFN1/2; OPA1; DRP1; MFF	Network remodeling, content mixing and segregation	Fragmentation is not a specific indicator of damage or clearance.	[23,33,45]
Biogenesis	PGC-1α; NRF1; TFAM	Renewal of mitochondrial proteins and genomes	Increased mass may reflect biogenesis, reduced removal, or both.	[24,25,30]
Ubiquitin-linked mitophagy	PINK1; Parkin; PARL; USP30	Damage recognition and regulation of mitochondrial ubiquitination	Recruitment and marker abundance do not establish completed degradation.	[51,54,56]
Receptor-mediated mitophagy	BNIP3; BNIP3L/NIX; FUNDC1	Receptor-mediated capture by autophagic machinery	Pathway contribution depends on tissue, developmental stage, and stress.	[49,50]
Alternative mitophagy	ULK1; RAB9	Alternative autophagic delivery during selected cardiac stresses	Evidence from pressure overload is indirect for inherited disorders.	[53]
Lysosomal completion	RAB7; LAMP proteins; TFEB; lysosomal hydrolases	Fusion, acidification and breakdown of delivered cargo	Normal lysosome number does not establish normal clearance; pH affects reporter readouts.	[29,31,54]

Mitochondrial permeability transition is discussed as an injury mechanism in Section 2.3 and Section 4.2, rather than classified as a quality-control process. LONP1, mitochondrial Lon peptidase 1; YME1L, YME1-like ATPase 1; OMA1, OMA1 zinc metallopeptidase; MFN, mitofusin; OPA1, optic atrophy protein 1; DRP1, dynamin-related protein 1; MFF, mitochondrial fission factor; PGC-1α, peroxisome proliferator-activated receptor γ coactivator 1α; NRF1, nuclear respiratory factor 1; TFAM, mitochondrial transcription factor A; TFEB, transcription factor EB. PINK1, PTEN-induced kinase 1; PARL, presenilin-associated rhomboid-like protein; USP30, ubiquitin-specific peptidase 30; BNIP3, BCL2-interacting protein 3; BNIP3L/NIX, BCL2-interacting protein 3-like; FUNDC1, FUN14 domain-containing protein 1; ULK1, Unc-51-like autophagy-activating kinase 1; RAB7/9, Ras-related proteins Rab-7/9; LAMP, lysosome-associated membrane protein.

**Table 2:** Disease- and gene-specific evidence relating mitochondrial defects to mitophagy.

Disease/Gene	Primary Defect and Cardiac Phenotype	Model	Mitophagy-Related Observation	Assay and Inference	Ref.
Barth/*TAFAZZIN*	Cardiolipin remodeling; dilated, hypertrophic, or noncompaction phenotypes	Tafazzin-deficient mouse embryonic fibroblasts	Reduced stress-induced mitophagosome formation	Colocalization/initiation assays. Noncardiac cellular evidence; not an in vivo cardiac degradation rate.	[26]
Barth/*TAFAZZIN*	Cardiolipin remodeling; cardiac-relevant cellular model	Isogenic human iPSC cardiomyocytes	Blunted CCCP-induced delivery to lysosomes	Mtphagy Dye–Lyso Dye colocalization. Direct delivery-associated assay; no complete degradation-rate measurement.	[27]
Barth/*Taz*	Cardiolipin remodeling; strain-dependent cardiomyopathy	*Taz*-knockout mice and isolated adult cardiomyocytes	Delivery-associated signal decreased in CAST/F1 and increased in A/J/F1 backgrounds	pH-sensitive dye plus cardiac phenotype. Context-dependent delivery readout, not proof of a universal flux defect.	[28]
Barth/*Taz*	Cardiolipin remodeling; dilated cardiomyopathy	*Taz*-knockdown mice; companion fibroblast experiments	Rapamycin improved cardiac function and autophagic/lysosomal findings	Cardiac immunoblots and ultrastructure; fibroblast colocalization. Cardiac degradation flux not directly quantified; intervention is pleiotropic.	[29]
Friedreich ataxia/*Fxn*	Iron–sulfur cluster and iron-homeostasis defect; hypertrophic cardiomyopathy	Muscle creatine kinase conditional knockout mice; human heart sections	Mouse findings supported increased autophagic and mitochondrial-substrate turnover	Bafilomycin-sensitive LC3/p62 and MFN1 accumulation provides dynamic evidence with limited mitochondrial specificity. Human data are static markers.	[30]
Friedreich ataxia/*Fxn*	Frataxin deficiency; cardiac hypertrophy	Heart-specific deficient mice	Lysosomal dysfunction and impaired-clearance interpretation	p62/Parkin and mTOR–TFEB/lysosomal findings. Not a mitochondria-specific degradation-rate assay.	[31]
Friedreich ataxia/frataxin	Mitochondrial stress; no myocardial phenotype tested	*Drosophila* glia	Increased delivery-associated reporter signal	mtRosella reporter; not a complete degradation-rate assay. Noncardiac, cross-species evidence; indirect for human cardiomyopathy.	[32]
*FARS2*	Mitochondrial translation; hypertrophy, dilatation and heart failure	Conditional mouse models; neonatal rat cardiomyocytes; human tissue	Stage-related marker changes; increased early cellular autophagy	Bafilomycin and tandem LC3 support general autophagic flux in cultured cells. Cardiac mitochondrial-marker findings do not alone measure selective flux.	[33]
*CHCHD10*	IMS proteostasis and cristae/stress signaling; murine cardiomyopathy	p.S55L knock-in mice	Mitophagy not established as the mediator	Aggregation, respiration and stress-pathway intervention. No direct mitophagy-flux evidence in the cited studies.	[46,47]
*AGK*; *ACAD9*; selected mtDNA variants	Import, assembly, or OXPHOS defects; variable cardiomyopathy	Disease-specific biochemical, clinical, or cellular reports	No adequate cardiac mitophagy-flux evidence established in the selected literature	Evidence gap, not evidence that mitophagy is normal. Each gene requires separate validation.	[1,13,58,60,61]
*CPT2*—comparator	Fatty acid oxidation defect; severe murine cardiomyopathy	Cardiomyocyte-specific *Cpt2* knockout ± *Usp30* deletion	Reduced mt-Keima delivery signal; genetic rescue of signal and cardiac outcomes	Mitochondria-specific lysosomal-delivery reporter plus intervention. Direct within this model; indirect for other inherited mitochondrial disorders.	[56]
HFpEF—comparator	Acquired metabolic/hemodynamic stress	Experimental murine HFpEF	Blunted cardiac mitophagy response	Reporter-based cardiac evidence in acquired disease; not a primary inherited mitochondrial cardiomyopathy.	[63]

Direct lysosomal delivery is distinguished from a dynamic degradation-rate measurement. pH-sensitive reporters and dyes require lysosomal pH and time-course controls; LC3/p62 changes can reflect synthesis, recruitment, or impaired degradation. “No adequate evidence” describes the selected literature and is not a claim that no study exists. iPSC, induced pluripotent stem cell; CCCP, carbonyl cyanide m-chlorophenylhydrazone; IMS, intermembrane space; HFpEF, heart failure with preserved ejection fraction. See Section 2.5 for assay interpretation. OXPHOS, oxidative phosphorylation; mtDNA, mitochondrial DNA; LC3, microtubule-associated protein 1 light chain 3; p62, sequestosome 1; MFN1, mitofusin 1; mTOR, mechanistic target of rapamycin; TFEB, transcription factor EB. Gene symbols are italicized; protein names are in roman type. The gray header background is for readability only and has no scientific meaning.

**Table 3:** Candidate biomarkers and cardiac assessments: defensible uses and limitations.

Measure	Biological Signal	Defensible Use	Principal Limitation	Ref.
Lactate ± lactate/pyruvate	Systemic redox and metabolic disturbance	Supportive biochemical assessment	Collection-dependent; not cardiac-specific; interpret the ratio in the appropriate biochemical context.	[5]
GDF-15	Systemic stress response	Diagnostic support; exploratory longitudinal assessment	Not cardiac-specific or a validated cardiac efficacy surrogate; affected by age and comorbidity.	[64,67]
FGF-21	Systemic, often muscle-related mitochondrial stress	Support for selected translation/maintenance disorders	Variable sensitivity; not a validated marker of myocardial progression or mitophagy.	[65,67]
Cell-free mtDNA	Release from uncertain tissue sources	Exploratory injury marker	Preanalytical and platelet effects; Fabry evidence is indirect; not myocardial heteroplasmy or flux.	[66]
hs-troponin/NT-proBNP	Myocardial injury/wall stress	Conventional cardiac assessment and serial follow-up	Neither identifies the mitochondrial mechanism; renal function and other factors affect interpretation.	[1,6]
Echocardiography/strain	Structure and ventricular function	Cardiac surveillance and phenotype assessment	Loading and technical variability; no direct mitochondrial turnover measurement.	[1,6]
CMR LGE and T1/ECV	Tissue characteristics including fibrosis	Phenotyping and exploratory trial stratification	No validated mitochondrial-disease cutoff for irreversible injury; feasibility varies.	[1,6,59]
31P-MRS PCr/ATP	Myocardial energetic state	Specialized mechanistic studies	Not a direct measure of spare respiratory capacity, mitophagy, or clinical benefit; access limited.	[13]

hs, high sensitivity; NT-proBNP, N-terminal pro-B-type natriuretic peptide; ECV, extracellular volume; PCr, phosphocreatine. A change in a systemic biomarker should not be attributed to cardiac improvement without concordant cardiac evidence. GDF-15, growth differentiation factor 15; FGF-21, fibroblast growth factor 21; mtDNA, mitochondrial DNA; CMR, cardiac magnetic resonance; LGE, late gadolinium enhancement; 31P-MRS, phosphorus-31 magnetic resonance spectroscopy; ATP, adenosine triphosphate.

**Table 4:** Mechanism-directed therapies by evidence stage and the remaining cardiac questions.

Strategy	Cell/Animal Evidence	Human Evidence or Indication	Main Unresolved Issue	Ref.
Elamipretide	Membrane and mitochondrial-marker effects in tafazzin-deficient models	Randomized primary endpoints not met; uncontrolled extension signals. Accelerated approval for muscle strength in Barth syndrome ≥30 kg, based on knee-extensor strength	Cardiac disease modification and clinical-event benefit unconfirmed; no general mitochondrial cardiomyopathy indication.	[17,68,69,70]
AAV-TAZ	Prevention/reversal of murine heart failure; mitochondrial and cardioskeletal improvement	No established human cardiac efficacy in the cited evidence	Systemic AAV immune/liver/complement risks; tissue coverage, dose, durability, and redosing.	[18,19,71]
AAV-FXN	Murine cardiac rescue; toxicity with excessive expression	2026 pooled open-label phase 1/2 evidence in 17 adults; exploratory cardiac signals, not established efficacy	Systemic AAV risks plus frataxin overexpression; myocarditis and immunosuppression risks; durability.	[20,21,22,71,72,73]
mtDNA base editing	Sequence editing in cells; delivery to mouse postmitotic tissues	No established human cardiac efficacy	Editing scope, bystander/off-target changes, distribution, and sufficient myocardial correction.	[74,75,76]
Riboflavin/matched cofactors	Biochemical rationale in specific defects	Observational response in a subset with ACAD9 deficiency	Variant- and disease-specific response; controlled cardiac evidence limited.	[5,58]
Idebenone/quinones	Electron-transfer and redox rationale	Early positive cardiac reports; negative larger randomized Friedreich ataxia cardiac study	No class-wide efficacy; target engagement and controlled outcomes required.	[77,78]
NAD+/substrate strategies	Context-dependent animal and metabolic evidence	No established class-wide cardiac efficacy	Blocked pathway, toxic intermediates, and extracardiac effects must be considered.	[30,47]
USP30 targeting	Genetic rescue in *Cpt2*-deficient mouse hearts with mt-Keima readout	No established efficacy for inherited mitochondrial cardiomyopathy	Genetic deletion is not drug efficacy; extension to other genotypes and degradation completion remain unproven.	[56]
mTOR/broader turnover modulation	Rapamycin cardiac benefit in *Taz* knockdown; broader network rescue in FARS2 models	No established disease-specific mitophagy treatment	Pleiotropy and systemic effects; risk of excessive mitochondrial depletion; direct flux and causal testing needed.	[29,33]

The table summarizes the strongest evidence cited for each strategy; it does not imply equivalence between evidence stages or rank comparative efficacy. Approved muscle-strength improvement is not an approved cardiac outcome. AAV, adeno-associated virus; TAZ, historical symbol for TAFAZZIN (tafazzin); FXN, frataxin; mtDNA, mitochondrial DNA; ACAD9, acyl-CoA dehydrogenase family member 9; NAD+, oxidized nicotinamide adenine dinucleotide; USP30, ubiquitin-specific peptidase 30; Cpt2, carnitine palmitoyltransferase 2; mTOR, mechanistic target of rapamycin; FARS2, mitochondrial phenylalanyl-tRNA synthetase 2.

## Data Availability

No new data were created or analyzed in this study. Data sharing is not applicable to this article.

## Abbreviations

Abbreviation	Definition
31P-MRS	phosphorus-31 magnetic resonance spectroscopy
AAV	adeno-associated virus
ACAD9	acyl-CoA dehydrogenase family member 9
ADP	adenosine diphosphate
AGK	acylglycerol kinase
ATP	adenosine triphosphate
ATF3	activating transcription factor 3
BNIP3	BCL2 interacting protein 3
BNIP3L/NIX	BCL2 interacting protein 3 like/NIP3-like protein X
CCCP	carbonyl cyanide m-chlorophenylhydrazone
CHCHD10	coiled-coil-helix-coiled-coil-helix domain containing 10
CMR	cardiac magnetic resonance
CPT2	carnitine palmitoyltransferase 2
CoQ	coenzyme Q
CRISPR	clustered regularly interspaced short palindromic repeats
DdCBE	DddA-derived cytosine base editor
DELE1	DAP3 binding cell death enhancer 1
DRP1	dynamin-related protein 1
ECG	electrocardiogram
ECV	extracellular volume
FARS2	phenylalanyl-tRNA synthetase 2, mitochondrial
FDA	US Food and Drug Administration
FGF-21	fibroblast growth factor 21
FUNDC1	FUN14 domain-containing protein 1
FXN	frataxin
GDF-15	growth differentiation factor 15
HFpEF	heart failure with preserved ejection fraction
HRI	heme-regulated inhibitor kinase
hs	high sensitivity
IMM	inner mitochondrial membrane
IMS	intermembrane space
iPSC	induced pluripotent stem cell
LC3	microtubule-associated protein 1 light chain 3
LGE	late gadolinium enhancement
LONP1	Lon peptidase 1, mitochondrial
MFF	mitochondrial fission factor
MFN1/2	mitofusin 1/2
mPTP	mitochondrial permeability transition pore
mtDNA	mitochondrial DNA
mTOR	mechanistic target of rapamycin
NAD+	oxidized nicotinamide adenine dinucleotide
NRF1	nuclear respiratory factor 1
NT-proBNP	N-terminal pro-B-type natriuretic peptide
OMM	outer mitochondrial membrane
OPA1	optic atrophy protein 1
OXPHOS	oxidative phosphorylation
p62	sequestosome 1 (SQSTM1)
PARL	presenilin-associated rhomboid-like protein
PCr	phosphocreatine
PGC-1α	peroxisome proliferator-activated receptor γ coactivator 1α
PINK1	PTEN-induced kinase 1
ROS	reactive oxygen species
TAFAZZIN/TAZ	tafazzin (historical gene symbol TAZ)
TFAM	mitochondrial transcription factor A
TFEB	transcription factor EB
TIM22	translocase of the inner mitochondrial membrane 22
ULK1	Unc-51-like autophagy activating kinase 1
USP30	ubiquitin-specific peptidase 30
YME1L	YME1-like ATPase 1
